# Supplementary figures and images for: RHS-elements function as type II toxin-antitoxin modules that regulate intra-macrophage replication of Salmonella Typhimurium
Source: PLoS Genet. 2020 Feb 13;16(2):e1008607. doi: 10.1371/journal.pgen.1008607 (PMC7043789; doi:10.1371/journal.pgen.1008607)

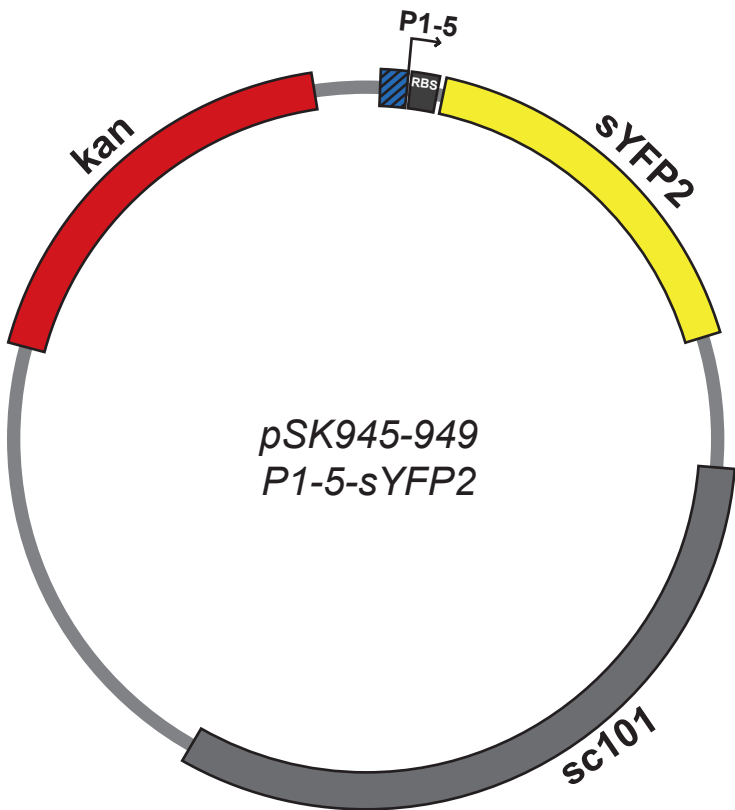

Supplement: S1 Fig — (PDF) [file pgen.1008607.s001.pdf]

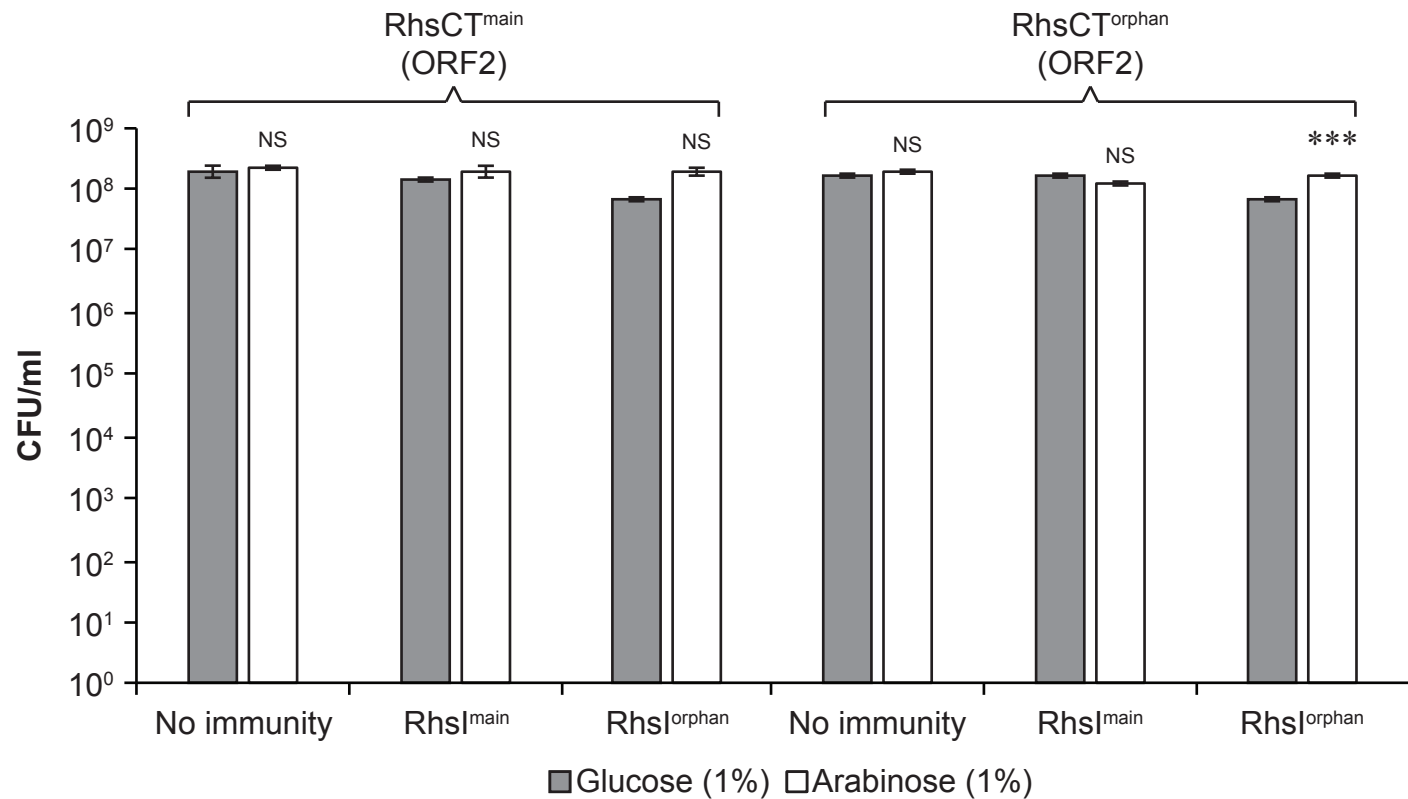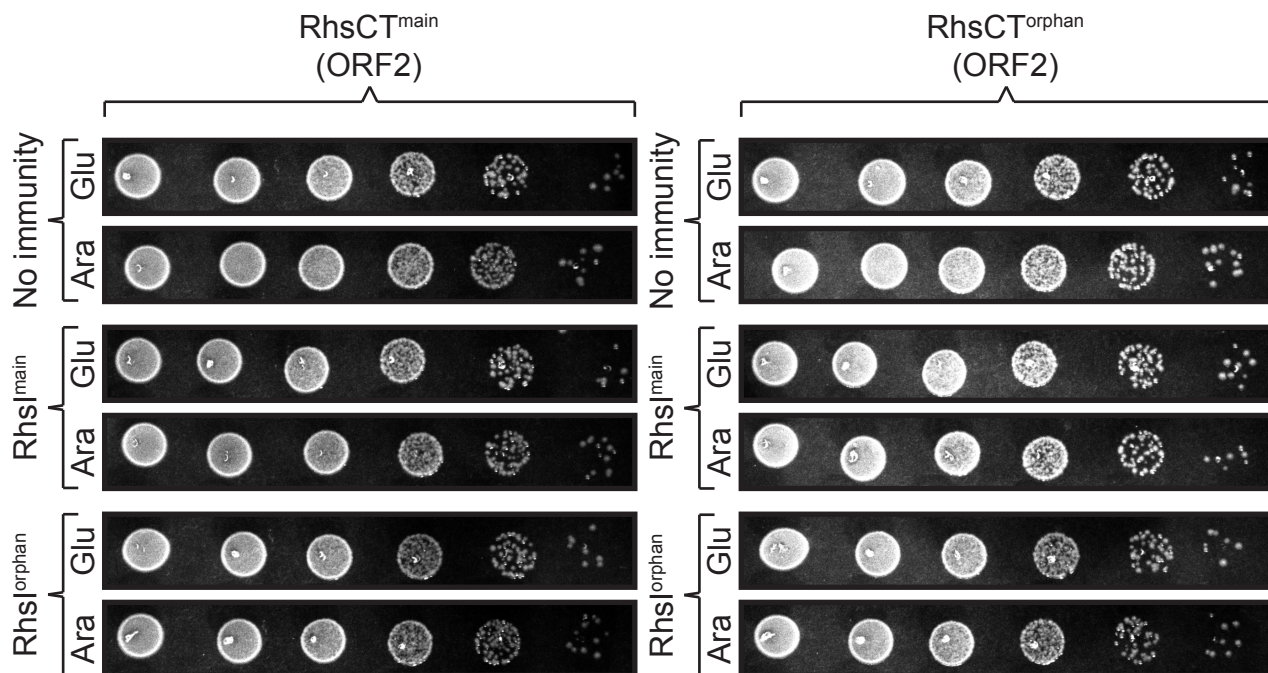

Supplement: S3 Fig — CFU counts from transformations of plasmids encoding arabinose inducible rhs-CTmain/rhs-CTorphan (ORF2) into NEB 5-α harboring no immunity or rhsImain/rhsIorphan on a plasmids grown in either 1% glucose or 1% arabinose. n = 3, Error-bars are SEM. Statistical significance was determined using two-tailed students t-test where *** = P<0.005. (PDF) [file pgen.1008607.s003.pdf]

**A)**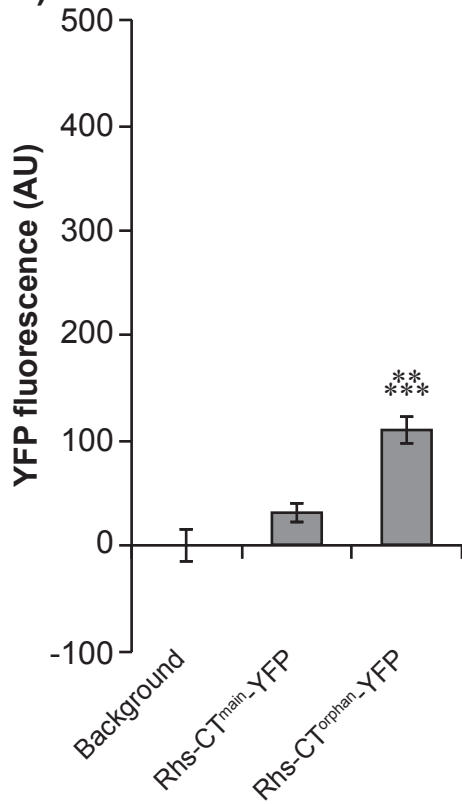**B)**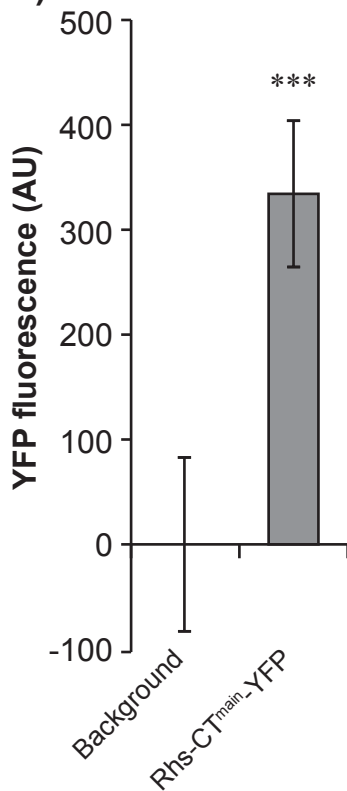

Supplement: S4 Fig — Single molecule fluorescence of strains with translational fusions of Rhs-CTmain and Rhs-CTorphan ORF1’s (CTG) to sYFP2. Strains were grown in M9-glucose to reduce background fluorescence. A) YFP fluorescence (au). B) Repeated experiment at higher laser voltage. Error-bars are SEM. Statistical significance was determined using two-tailed students t-test where *** = P<0.001 and ***** = P<0.00001. (PDF) [file pgen.1008607.s004.pdf]

0 h

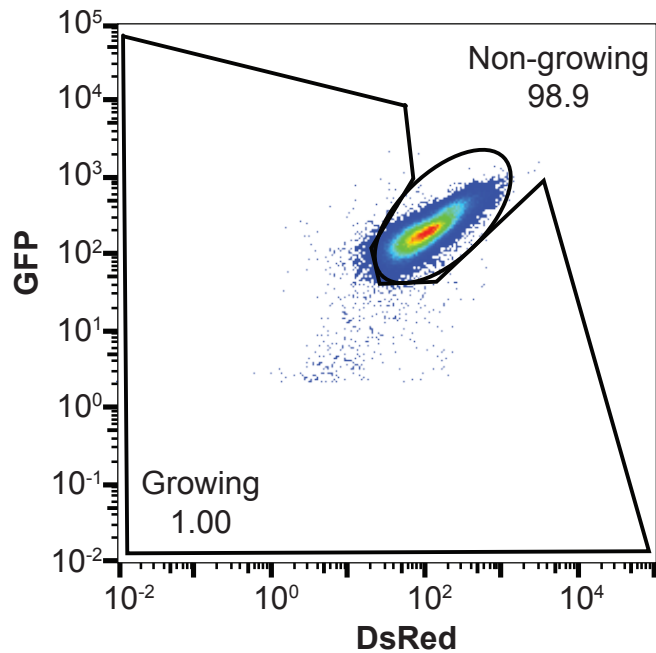

16 h

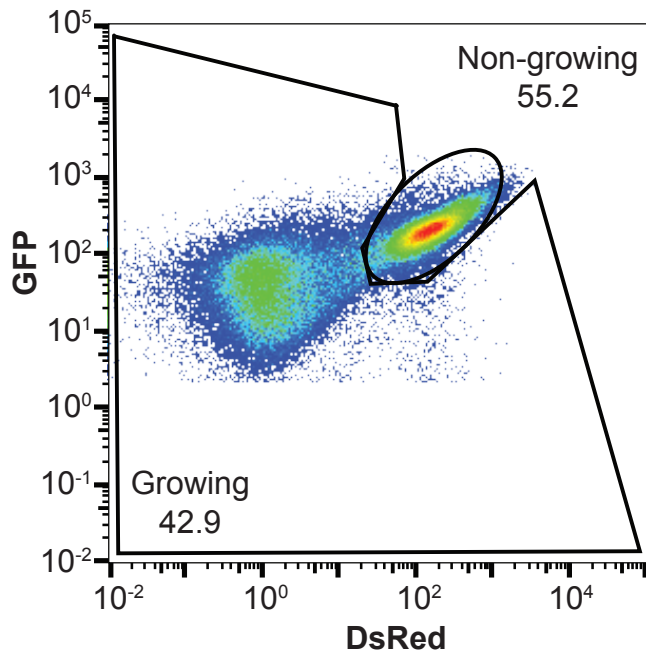

Supplement: S5 Fig — Representative graph of flowcytometric data used to identify the dividing population of bacteria after 16h of growth in RAW264.7 macrophages. The growing population is determined as the cells where the dsRed fluorescent signal is decreased to levels below half of the mean fluorescent signal of the non-growing population. (PDF) [file pgen.1008607.s005.pdf]
