# Supplementary material for: RHS-elements function as type II toxin-antitoxin modules that regulate intra-macrophage replication of Salmonella Typhimurium
Source: PLoS Genet. 2020 Feb 13;16(2):e1008607. doi: 10.1371/journal.pgen.1008607 (PMC7043789; doi:10.1371/journal.pgen.1008607)
Supplement: S2 Fig — Overview of the A) P2A or B) P2B promoter sequences. -35 (yellow) and -10 (blue) regions were predicted using the softberry BPROM software. Transcription start sites (TSS´s) (bold and underlined) were determined using C) 5’RACE. Potential start codons are in green, RBS’s in orange and in-frame stop codons in pink. (PDF) [file pgen.1008607.s002.pdf]

A) $P2^{A}$: *rhs-CT*^{main}

TTTGGAGAGAATGCGGCGGACATCAGCAACAGCGGGGCGTACTTTCACCAGCCGCTGCG

-35 **-10** **TSS**

CGGCTGCCGGGGCAGTATTTTGACGACGAGACAGGGCTGCATTACAATCTGTTC<u>**A**</u>GATA

RBS **Start: ORF1**

TTATGCACCGGAGTGTGGACGGTTTGTCAGTCAGGATCCGATCGGG**CTG**AGGGGCGGGT

TAAACCTTTATCAGTATGCGCCAAATCCTCTCAAATATATAGACCCACTTGGTTTAACC

Start: ORF2

GCGACTGTTGGGCGATGG**ATG**GGGCCTGCGGAATATCAGCAAATGCTTGATACTGGGAC

AGTAGTACAAAGTTCAACAGGGACAACTCATGTTGCCTACCCTGCTGATATAGATGCTT

TTGGTAAGCAAGCAAAAAATGGTGCT**ATG**TATGTTGAATTTGATGTGCCTGAAAAATCA

-35 **-10**

TTAGTACCTACAAATGAAGGATGGGCAAAAATAGTAGGGCCAGATTCTATCGAAGGGCG

ATTAGCTAAACGCAAAGGTTTGCCTGTTCCTGAAATGCCAACAGCAGAAAACATAACTG

RBS

TAAGGGGCGAGAAAATTAATGGGGAAGTTGAAGCAAA**ATG**C**TAA**

B) $P2^{B}$: *rhs-CT*^{orphan}

TTTGGAGAAAATGCGGCGGACATCAGCAACAGCGGGGCGTACTTTCACCAGCCGCTGCG

-35 **-10** **TSS**

GCTGCCGGGGCAGTATT**TTGACG**ACGAGACAGGGCTG**CATTACAAT**CTGTTC<u>**A**</u>GATATT

RBS **Start: ORF1**

ATGCACCGGAGTGTGGACGGTTCGTCAGTCAGGATCCGATTGGG**CTG**GCGGGGGGG**CTG**

AATCTTTACCAGTATGCGCCTAATCCGATTAGATGGATCGATCCTTTAGGACTTGCTAT

C**CTG**GAGCATCAATCTAATTTTGATGCGGCAAGGAGAACCGGATTTGAAAATGCGGGT**A**

Start: ORF2

TGACAAACCCTGAGGATGTCACTTTCTCGAAAGTCGATCCCAAAACTGGTACTGTTGTT

GAGTTTAAAGGTCCAAATGGGGCTAAAGTTGCTTATGATGCACCTCATGCAGAT**ATG**GA

TGTGACAGCAGGGCATGATAAACCACATGTTGGTTGGCAATCCGCAGGAAAAAGAGGTT

CCGGAGGAGCTAATAGAGGTAATATTACTTATGATGGCCCACAACATCCGCATCGCTCT

GACTCTAAGGGAGATGATAAATGT**TAA**
