# Supplementary material for: RHS-elements function as type II toxin-antitoxin modules that regulate intra-macrophage replication of Salmonella Typhimurium
Source: PLoS Genet. 2020 Feb 13;16(2):e1008607. doi: 10.1371/journal.pgen.1008607 (PMC7043789; doi:10.1371/journal.pgen.1008607)
Supplement: S1 Text — Supplementary methods and Tables A-C. (PDF) [file pgen.1008607.s007.pdf]

S1 text

Supplementary information for:

Title: RHS-elements function as Type II Toxin-Antitoxin modules that regulate intra-macrophage replication of *Salmonella* Typhimurium

Magnus Stårsta [†], Disa L Hammarlöf[†], Marcus Wäneskog, Susan Schlegel, Feifei Xu, Arvid Heden Gynnå, Malin Borg, Sten Herschend and **Sanna Koskiniemi*.**

Affiliations:

Department of Cell and Molecular Biology, Uppsala University, 75124 Sweden.

* Correspondence to: sanna.koskiniemi@icm.uu.se

† These authors contributed equally to this work.

This document contains:

Supplementary methods

Supplementary Tables A-C

Supplementary methods

Strain and plasmid constructions

Plasmid constructions

Cloning of transcriptional fusions of rhs promoters with YFP

P1-5 promoters sequences were amplified using oligos SK393-SK402 (Table S1) and cloned into pEH167 using XhoI and BamHI (Thermo fisher). pEH167 was generous gift from E. Holmqvist and was constructed previously by replacing GFP with the yellow fluorescent protein (YFP) variant Venus in the pUA66 plasmid [1]. In short, the pUA66 plasmid was linearized by PCR using oligos EHO615-EHO616, cut with NheI and SbfI, and ligated to a NheI/SbfI digested PCR product of the *yfp* (venus) amplified using oligos EHO566-EHO691. To include binding sites for as many as possible regulators, the promoters were defined as either 100 ($P2^{A-B}$-P5) or 200bp (P1) upstream of the predicted TSS. Gel-purified vector backbone and insert DNA digestion products were ligated with T4 DNA ligase (Thermo Fisher) and transformed into NEB® 5-alpha Competent *E. coli* (High Efficiency) (New England Biolabs). Successful clones were validated by PCR and sequencing (Eurofins).

Cloning of rhs-CT's under arabinose inducible promoters

The ORFs encoding *rhs-CT*^{main} and *rhs-CT*^{orphan} were amplified using oligos SK713/759 and SK715/761 (for the longer constructs starting from CTG) or SK740/759 and SK743/761 (for the shorter constructs starting from ATG). PCR products of the correct size were purified and cloned in pCH450 using EcoRI and XhoI. Constructs were verified by PCR and sequencing.

Cloning of rhsI's under arabinose inducible promoters

A Kanamycin resistance gene was amplified by PCR from pKD4 [2] using oligos SK313/16 (*rhsI*^{main}) and SK314 /22 (*rhsI*^{orphan}) (Table S1). The amplified fragments were inserted in the

S. Typhimurium genome using lambda red recombination, generating an HA-tag followed by a stop codon (TAA) fusion to the end of either immunity gene. The *rhsI*-HA-STOP constructs were amplified from genomic DNA by PCR using primers SK1405/1407 and SK1406/1407 and cloned into /pBAD33 [3] using PstI and HindIII.

Cloning of rhsCT+I^{orphan} under arabinose inducible promoters

rhsCT+I^{orphan} [P2-ORF1] was amplified from the *Salmonella* chromosome using primers 715/1319 or 715/1407 for HA-tagged *rhsI.* PCR products of the correct size were purified and cloned in pBAD30/pBAD24 using EcoRI and HindIII. Constructs were verified by PCR and sequencing using oligos 898/87.

Chromosomal constructs

Construction of translational fusions and genetic knock-outs

Translational fusions to Rhs-CT^{main} and Rhs-CT^{orphan} were constructed by amplifying YFP-kan cassette from SK442 using oligos SK745/186 and SK749/186 respectively. Purified PCR products were used for lambda red recombination as described previously [2]. Constructs were verified by PCR and sequencing using oligos SK1/336 or SK11/336 for the Rhs-CT^{main} and Rhs-CT^{orphan} constructs respectively. Deletions in the *rhs*-locus were made by amplifying kanamycin (for toxin knockouts) or chloramphenicol (for immunity knock outs) resistance genes from pKD4/pKD3 respectively using oligos: SK56/57 (*rhs-CT*^{main}), SK56/16 (*rhs-CT-I*^{main}), SK27/324 (*rhs-CT*^{orphan}), SK27/186 (*rhs-CT-I*^{orphan}) and SK15/186 (*rhs*^{complete}). Transformants were selected on LB plates containing appropriate antibiotic and screened by PCR and sequencing using oligos SK1/12 (*rhs-CT*^{main}), SK1/6 (*rhs-CT-I*^{main}), SK11/14 (*rhs-CT*^{orphan}), SK1/336 (*rhs-CT-I*^{orphan}) and SK9/336 (*rhs*^{complete}) respectively. The *rhs*-locus was reinserted into Δ*rhs*^{complete}, by duplication-insertion engineering [4]. In short, a duplication of

the *rhs*-locus was created by amplifying a *cat-sacB* cassette using oligos SK176/177 and inserting the amplified PCR product in the genome of *S.* Typhimurium LT2 using lamda red recombineering. The stable duplication was moved into SK3322 (Δ*rhs*^{complete}) with P22 transduction. The duplication was allowed to segregate during overnight growth in no salt LB and segregants were selected by plating on LB plates lacking NaCl and supplemented with 5% sucrose. Presence of the *rhs*-locus was verified by PCR using oligos SK1/4 and SK1/6.

Expression of translational fluorescent protein reporters using single cell microscopy

Over-night cultures were subcultured in 1:1000 in M9Glu media (1x M9 salts, 0.4% glucose, 2 mM $MgSO_4$, 100 µM $CaCl_2$ and 1% cas-amino acids) and grown for 4 hours to reach exponential phase. One microliter bacterial suspension was added onto 2% agarose in M9Glu pads. The cells were imaged in fluorescence (to detect cellular YFP expression levels) and bright field channels (to locate the cells for data analysis) using a Nikon Ti-E microscope with a Nikon Plan Apo Lambda 100x Oil immersion objective (NA 1.45), an Andor iXon3 897 EMCCD camera, and a stage incubator set to 37°C. Fluorescent microscopy employed a 561nm laser (Genesis CX STM, Coherent) with an exposure time of 500 ms. More than 100 cells per strain per replicate were randomly selected in bright field. Average cell fluorescence was collected from a 49 px^2 (0.123 μm^2) square region in the middle of the selected cell. Background fluorescence was measured from a strain carrying the same YFP gene without translation start. Data analysis is performed by custom developed algorithm in MATLAB.

Table A. Oligos used in this study.

Oligo	Sequence (5'-3')	Comment
1	ACAGAAGCGGTTACCTGAC	Forward primer used to verify presence of the *rhs* locus
3	CTACAAATGAAGGATGGGCA	Forward primer used for rt-qPCR of *rhsCT (main)*
4	CTCGCCCCTTACAGTTATGT	Reverse primer used for rt-qPCR of *rhsCT (main)*
5	ACGGTTCGTCAGTCAGGAT	Forward primer used for rt-qPCR of *rhsCT (orphan)*
6	AGGGTTTGTCATACCCGCAT	Reverse primer used for rt-qPCR of *rhsCT (orphan)*
7	TACCGAACATCACGCCAATC	Forward primer used for rt-qPCR of *recA*
8	GTATGATGAGCCAGGCGATG	Reverse primer used for rt-qPCR of *recA*
9	TGGTCTTCACCCTGACCAGT	Reverse primer used to confirm Δ*rhs (complete)* construct
11	GTGGGTGAGCAAACATACTG	Forward primer used for verification of the presence of *rhsI (main)*
12	CATACAGCTTAGGTCATCCCA	Reverse primer used to confirm Δ*rhsCT (main)* construct
14	TATCCAGAATTATCGGTATAAG	Reverse primer used to confirm Δ*rhsCT (orphan)* construct
15	TCAGCCGGATAAAAACGAATAATCACACGGAGGTGTGACCtgtaggctggagctgcttc	Forward primer used to knock out *rhs (delivery)* using lambda red recombination
16	TAAAACAGCCGGGAAGAGTTAACTTCTTACCCGGCCAACTcatatgaatatcctcctta	Reverse primer used to knock out *rhsI (main)* using lambda red recombination
22	CACTCGCGCTTTACTTAATGGTTGAGCATACAATCAAATCcatatgaatatcctcctta	Reverse primer used to knock out *rhsI (orphan)* using lambda red recombination
27	CGGAGTGTGGACGGTTCGTCAGTCAGGATCCGATTGGGCTtgtaggctggagctgcttc	Forward primer used to knock out *rhsCT (orphan)* or *rhsI (orphan)* using lambda red recombination
56	GCGACTGTTGGGCGATGGATGGGGCCTGCGGAATATCAGCtgtaggctggagctgcttc	Forward primer used to knock out *rhsCT (main)* or *rhsI (main)* using lambda red recombination
57	ACAATTTAAATTTATTTAGCATTTTGCTTCAACTTCCCCcatatgaatatcctcctta	Reverse primer used to knock out *rhsCT (main)* using lambda red recombination
87	gcgtttcacttctgagttcg	Reverse primer to confirm */pBAD* constructs
176	AGCCTGCCAGCCAGGTGTTCCAGAGTAGGTCTGCTTTATCCATATGAATATCCTCCTTAGTTCC	Forward primer used to create duplication of the *rhs*-locus
177	CTAGCCGAAGATGGCGCGATTGTCTGGCAGGGGAAACAGCTGTAGGCTGGAGCTGCTTC	Reverse primer used to create duplication of the *rhs*-locus

186	GCTGTTTCCCCTGCCAGACAATCGCGCCATCTTCGGCTAGCATATGAATATCCTCCTTAGTTCC	Reverse primer used to insert sYFP2-kan after *rhsI (orphan)* using lamda red recombination
206	ATCTGATTACCTGGCGGACACTAAACTAAGAGAGAGCTCTtgtaggctggagctgcttc	Forward primer used to knock out *lon* using lambda red recombination
207	TGCCAGCCCTGTTTTTATTAGCGCTATTTGCGCGAGGTCAcatatgaatatcctcctta	Reverse primer used to knock out *lon* using lambda red recombination
208	GATACCATGTACGATTTGCC	Forward external primer used to confirm strain constructions in *lon*
209	CAAAGCCTACCAGTGCAAC	Reverse external primer used to confirm strain constructions in *lon*
313	ACTTCTGGATATATTTGATGATTTTATGATAAGTATTAAATACCCATACGATGTTCCAGATTACGCTTAATGTAGGCTGGAGCTGCTTC	Forward primer used to introduce HA-tag-STOP in *rhsI (main)* by lambda red recombination
314	AAATGATCATGGTGATTTGATTGTATGCTCAACCATTAAGTACCCATACGATGTTCCAGATTACGCTTAATGTAGGCTGGAGCTGCTTC	Forward primer used to introduce HA-tag-STOP in *rhsI (orphan)* by lambda red recombination
315	TCTCCTGCAACAGCCTGAAC	Reverse primer used to confirm strain constructions of *rhsI (main)*
324	ACATATTTGAATTTAACATTTATCATCTCCCTTAGAGTCcatatgaatatcctcctta	Reverse primer used to knock out *rhsCT (orphan)* using lambda red recombination
332	ATATCAGCAGGGTAGGCAAC	Used for 5'RACE in *rhsCT (main)*
362	TGGTACGACAGGGAAACGTA	Forward primer used for rt-qPCR of *rhs (delivery)*
363	CAATGTTGACAGGGAAGTGG	Reverse primer used for rt-qPCR of *rhs (delivery)*
387	CGCTTCAGCCATACTTTTCA	Forward primer to confirm */pBAD* constructs
388	GTCTCATGAGCGGATACATATTTG	Reverse primer to confirm */pBAD* constructs
393	ATATctcgagAACAAATTGCCGCCACCCAT	Forward primer with (XhoI) used for cloning of transcriptional fusion P1 (-200 nt from ORF *STM0291*) with YFP in */pEH167*
394	GCGCggatccTGGTCACACCTCCGTGTGATT	Reverse primer with (BamHI) used for cloning of transcriptional fusion P1 (-200 nt from ORF *STM0291*) with YFP in */pEH167*
395	ATATctcgagTTTGGAGAGAATGCGGCG	Forward primer with (XhoI) used for cloning of transcriptional fusion P2 (-100

		nt from ORF *STM0291*) with YFP in */pEH167*
396	GCGCggatccTGAACAGATTGTAATGCAGCCCT	Reverse primer with (BamHI) used for cloning of transcriptional fusion P2 (-100 nt from ORF *STM0291*) with YFP in */pEH167*
397	ATATctcgagGGTAAGCAAGCAAAAAATGGTGC	Forward primer with (XhoI) used for cloning of transcriptional fusion P3 (-100 nt from ORF *STM0291*) with YFP in */pEH167*
398	GCGCggatccTCTGGCCCTACTATTTTTGCC	Reverse primer with (BamHI) used for cloning of transcriptional fusion P3 (-100 nt from ORF *STM0291*) with YFP in */pEH167*
399	ATATctcgagTTGATGCGGCAAGGAGAAC	Forward primer with (XhoI) used for cloning of transcriptional fusion P4 (-100 nt from ORF *STM0291*) with YFP in */pEH167*
400	GCGCggatccCTCAACAACAGTACCAGTTTTGgg	Reverse primer with (BamHI) used for cloning of transcriptional fusion P4 (-100 nt from ORF *STM0291*) with YFP in */pEH167*
401	ATATctcgagAGTCAGGATCCGATTGGGC	Forward primer with (XhoI) used for cloning of transcriptional fusion P5 (-100 nt from ORF *STM0291*) with YFP in */pEH167*
402	GCGCggatccTGCTCCAGGATAGCAAGTCC	Reverse primer with (BamHI) used for cloning of transcriptional fusion P5 (-100 nt from ORF *STM0291*) with YFP in */pEH167*
411	TGAATCTGACGAACACGTTCACG	Forward primer used to verify DNA degradation after DNase treatment
412	CCGCTTCTCAACATACGCAACC	Reverse primer used to verify DNA degradation after DNase treatment
620	TTGTCCAGACTATAGTTCTCAGACATGACGCCGGGGATGCGGCTAATGTAGATCG	Forward primer used to replace *STM1553* with a *cat* marker using lambda red recombination
621	GTGAGCGATTATTGTCCAGTTTTCTCTGGGCCTGGTTCGCGAAATAAACGACCGG	Reverse primer used to replace *STM1553* with a *cat* marker using lambda red recombination
622	ACGGCTGCTGAAACGTTATC	Forward internal primer used to confirm strain constructions in *STM1553*
623	TATCAACAATTGCGACGGGG	Reverse internal primer used to confirm strain constructions in *STM1553*
624	TAGTTCTCAGACATGACGCC	Forward external primer used to confirm strain constructions in *STM1553*
625	TCCAGAGTGGTTCAATCCGT	Reverse external primer used to confirm strain constructions in *STM1553*

713	ATATgaattcCTGAGGGGCGGGTTA	Forward primer used for cloning *rhsCT (main)* internal ORF1 (CTG) into */pCH45*0 (EcoRI)
715	ATATgaattcCTGGCGGGGGGGCTGAAT	Forward primer used for cloning *rhsCT (orphan)* internal ORF1 (CTG) into */pCH450* or *pBAD* (EcoRI)
740	ATATgaattcATGGGGCCTGCGGAATAT	Forward primer used for cloning *rhsCT (main)* internal ORF2 (ATG) into */pCH450* (EcoRI)
743	ATATgaattcATGACAAACCCTGAGGATGT	Forward primer used for cloning *rhsCT (orphan)* internal ORF2 (ATG) into */pCH450* (EcoRI)
745	CGATCCTTTAGGACTTGCTATCCTGGAGCATCAATCTAATTTTGATGCGGCAAGGAGAACCGGATTTGAAAATGCGGGTATGGTTAGCAAGGGCGAA	Forward oligo used to create translational fusion of the *rhsCT (orphan)* to sYFP2
749	ACCCACTTGGTTTAACCGCGACTGTTGGGCGATGGATGGTTAGCAAGGGCGAA	Forward oligo used to create translational fusion of the *rhsCT (main)* to sYFP2
759	GCGCCTCGAGTTAGCATTTTGCTTCAACTT	Reverse primer used to clone *rhsCT (main)* products in */pCH450* (XhoI)
761	GCGCCTCGAGTTAACATTTATCATCTCCCTTAGAGTCAGAG	Reverse primer used to clone *rhsCT (orphan)* products in */pCH450* (XhoI)
882	TCATATTGTCCAGACTATAGTTCTCAGACATGACGCCGGGTGTAGGCTGGAGCTGCTTC	Forward primer used to replace *STM1553* with a *kan* marker using lambda red recombination
883	GTGAGCGATTATTGTCCAGTTTTCTCTGGGCCTGGCGCGCCATATGAATATCCTCCTTA	Reverse primer used to replace *STM1553* with a *kan* marker using lambda red recombination
898	ACTCTCTACTGTTTCTCCATACC	Forward primer to confirm */pBAD* constructs
1319	GCGCaagcttCTAAATATGGGCAACTATAT	Reverse primer used to clone *rhsCT+I (orphan)* into */pBAD* (HindII)
1405	ATATctgcagAATGGGGAAGTTGAAGCAAA	Forward primer used to clone *rhsI (main)-HA* into */pBAD3*3 (PstI)
1406	ATATctgcagGACTCTAAGGGAGATGATAA	Forward primer used to clone *rhsI (orphan)-HA* into */pBAD33* (PstI)
1407	GCGCaagcttTTAAGCGTAATCTGGAACAT	Reverse primer used to clone *rhsI-HA* into */pBAD* (HindII)
EHO-566	CCGGGCTAGCatggtgagcaagggcgag	Forward primer used to amplify *yfp* (venus) (NheI)
EHO-615	ccggGCTAGCCATATGTATATCTCCTTCTTAAATCTAGAGGATCCCCTC	Reverse primer used to linearize pUA66 (NheI)
EHO-616	CAGACCTGCAGGCATGCAAGC	Forward primer used to linearize pUA66 (SbfI)
EHO-691	CCGGGCCTGCAGGtcacaattccttgtacagctcgtc	Reverse primer used to amplify *yfp* (venus) (SbfI)

EHO-694	GGCAATTCCGACGTCTAAGAA ACC	Forward primer used to validate cloned inserts in */pEH167*
EHO-695	TCCTACTCAGGAGAGCGTTCA C	Reverse primer used to validate cloned inserts in */pEH167*
FS17	CGACTGGAGCACGAGGACAC TGA	RNA adaptor used for 5'RACE
FS354	GACACTGACATGGACTGAAG GAGTA	RNA adaptor (nested) used for 5'RACE

Table B. SNPs found in SK3322.

**Genetic location**	**Gene**	**Mutation**
325793	STM0333 (*vasK*)	Leu416Phe
328069	STM0333 (*vasK*)	Thr1175Met
332073	STM0338 (*vgrG*)	Thr506Ser

Table C. Bacterial strains and plasmids used in this study.

**Strain number**	**Description**	**Source**
SK1	*Salmonella enterica* serovar Typhimurium LT2, (*Sty* LT2)	Lab collection
SK502	*Sty* LT2, *zfa-9223::kan*(sw), *metA22, metE551, trpD2, ilv-452, leu-, pro-*(leaky), *hsdLT6, hsdSA29, hsdB, strA120* /pCH450::*rhsCT(orphan)*(H208A)	[5]
SK511	*Sty* LT2, *lon::kan*	This study
SK683	*Salmonella enterica* serovar Typhimurium 14028S (*Sty* 14028)	ATCC, CDC 6516-60
SK1092	*Sty* 14028 /pEH167 *rhsP1(STM0291)*:YFP promoter fusion 200 nt	This study
SK1093	*Sty* 14028 /pEH167 *rhsP2(STM0291_int)*:YFP promoter fusion 100 nt	This study
SK1094	*Sty* 14028 /pEH167 *rhsP3(STM0291_int)*:YFP promoter fusion 100 nt	This study
SK1095	*Sty* 14028 /pEH167 *rhsP4(STM0292_int)*:YFP promoter fusion 100 nt	This study
SK1096	*Sty* 14028 /pEH167 *rhsP5(STM0292_int)*:YFP promoter fusion 100 nt	This study
SK1097	*Sty* 14028 /pEH167	This study
SK1129	*Sty* 14028, Δ*rhsCT+I (orphan)*	This study
SK1722	*Sty* 14028, Δ*rhsCT+I (orphan)*, translational fusion P2-native RBS-CTG (main)-*syfp2*:kan	This study

SK1781	*Sty* 14028, Δ*rhsCT+I (orphan)*, translational fusion P2-native RBS-CTG (orphan)-*syfp2*:kan	This study
SK1788	*Sty* 14028, Δ*rhs-CT+I (main),* Δ*rhsCT+I (orphan)*, translational fusion P2-native RBS-first native ATG-*syfp2*::kan	This study
SK2080	*Sty* LT2 /pBAD33::*rhsI-HA (main)*	This study
SK2081	*Sty* LT2/ pBAD33::*rhsI-HA (orphan)*	This study
SK2786	*Sty* 14028 /pDiGc	This study
SK2787	*Sty* 14028, Δ*rhsCT+I (main)* /pDiGc	This study
SK2788	*Sty* 14028, Δ*rhsCT+I (orphan)* /pDiGc	This study
SK2789	*Sty* 14028*,* Δ*rhsCT+I (main),* Δ*rhsCT+I (orphan)* /pDiGc	This study
SK2790	*Sty* 14028, *Δrhs (complete)* /pDiGc	This study
SK2880	*Sty* LT2, *lon::kan* /pBAD33::*rhsI-HA (main)*	This study
SK2881	*Sty* LT2, *lon::kan* /pBAD33::*rhsI-HA (orphan)*	This study
SK3313	*Sty* 14028, *STM1553::terCATter*	This study
SK3318	*Sty* 14028, *STM1553::kan*	This study
SK3319	*Sty* 14028, Δ*rhsCT+I (main), STM1553::kan*	This study
SK3320	*Sty* 14028, Δ*rhsCT+I (orphan), STM1553::kan*	This study
SK3321	*Sty* 14028, Δ*rhsCT+I (main),* Δ*rhsCT+I (orphan), STM1553::kan*	This study
SK3322	*Sty* 14028, *Δrhs (complete), STM1553::kan*	This study
SK4261	*Sty* 14028 (ATCC), *STM1553::kan,* (*rhs* locus has been re-inserted into the Δ*rhs* (complete) background)	This study
SK4287	NEB 5-alpha /pDAL923	This study
SK4085	*Sty* LT2/ pBAD33::*rhsI-HA (orphan)*	This study
SK4086	*Sty* LT2, *lon::kan* /pBAD33::*rhsI-HA (orphan)*	This study
SK4288	NEB 5-alpha /pDAL938	This study
SK4289	NEB 5-alpha	This study
SK4251	NEB 5-alpha /pSK4251 (pBAD24::*rhsCT+I*(orphan)-HA [P2-ORF1])	This study
SK4292	NEB 5-alpha /pSK4251 (pBAD24::*rhsCT+I*(orphan)-HA [P2-ORF1]) /pCH450::rhsCT(orphan)(H208A)(sty)	This study

**Plasmid**	Genotype	**Origin**
pDAL923	pBR322::*rhsI (main)*	[5]
pDAL938	pBR322::*rhsI (orphan)*	[5]

pDiGc		[6]
pEH167	Empty vector	Erik Holmqvist
pSK502	pCH450::*rhsCT*(orphan)(H208A)	[5]
pSK945	pEH167 *rhsP1(STM0291*):YFP promoter fusion 200 nt	This study
pSK946	pEH167 *rhsP2(STM0291_int)*:YFP promoter fusion 100 nt	This study
pSK947	pEH167 *rhsP3(STM0291_int)*:YFP promoter fusion 100 nt	This study
pSK948	pEH167 *rhsP4(STM0292_int)*:YFP promoter fusion 100 nt	This study
pSK949	pEH167 *rhsP5(STM0292_int)*:YFP promoter fusion 100 nt	This study
pSK1913	pCH450::*rhsCT (main)* [P2-ORF1(CTG)]	This study
pSK1914	pCH450::*rhsCT (orphan)* [P2-ORF1(CTG)]	This study
pSK1915	pCH450::*rhsCT (main)* [P2-ORF2(ATG)]	This study
pSK1918	pCH450::*rhsCT (orphan)* [P2-ORF2(ATG)]	This study
pSK1969	pBAD33::*rhsI-HA (main)*	This study
pSK1970	pBAD33::*rhsI-HA (orphan)*	This study
pSK4251	pBAD24::rhsCT+I(orphan)-HA [P2-ORF1(CTG)]	This study

References

1. Zaslaver A, Bren A, Ronen M, Itzkovitz S, Kikoin I, Shavit S, et al. A comprehensive library of fluorescent transcriptional reporters for Escherichia coli. Nat Methods. 2006;3(8):623-8. Epub 2006/07/25. doi: 10.1038/nmeth895. PubMed PMID: 16862137.
2. Datsenko KA, Wanner BL. One-step inactivation of chromosomal genes in Escherichia coli K-12 using PCR products. Proceedings of the National Academy of Sciences of the United States of America. 2000;97(12):6640-5. Epub 2000/06/01. doi: 10.1073/pnas.120163297. PubMed PMID: 10829079; PubMed Central PMCID: PMC18686.
3. Guzman LM, Belin D, Carson MJ, Beckwith J. Tight regulation, modulation, and high-level expression by vectors containing the arabinose PBAD promoter. J Bacteriol. 1995;177(14):4121-30. Epub 1995/07/01. doi: 10.1128/jb.177.14.4121-4130.1995. PubMed PMID: 7608087; PubMed Central PMCID: PMCPMC177145.
4. Nasvall J, Knoppel A, Andersson DI. Duplication-Insertion Recombineering: a fast and scar-free method for efficient transfer of multiple mutations in bacteria. Nucleic Acids Res. 2017;45(5):e33. Epub 2016/12/03. doi: 10.1093/nar/gkw1078. PubMed PMID: 27899661; PubMed Central PMCID: PMCPMC5389514.
5. Koskiniemi S, Garza-Sanchez F, Sandegren L, Webb JS, Braaten BA, Poole SJ, et al. Selection of Orphan Rhs Toxin Expression in Evolved Salmonella enterica Serovar Typhimurium. PLoS Genet. 2014;10(3):e1004255. Epub 2014/03/29. doi: 10.1371/journal.pgen.1004255. PubMed PMID: 24675981.
6. Helaine S, Thompson JA, Watson KG, Liu M, Boyle C, Holden DW. Dynamics of intracellular bacterial replication at the single cell level. Proc Natl Acad Sci U S A. 2010;107(8):3746-51. Epub 2010/02/06. doi: 10.1073/pnas.1000041107. PubMed PMID: 20133586; PubMed Central PMCID: PMC2840444.
