# Supplementary material for: RHS-elements function as type II toxin-antitoxin modules that regulate intra-macrophage replication of Salmonella Typhimurium
Source: PLoS Genet. 2020 Feb 13;16(2):e1008607. doi: 10.1371/journal.pgen.1008607 (PMC7043789; doi:10.1371/journal.pgen.1008607)
Supplement: Attachment — Submitted filename: response to reviewers Plos genetics 191206.pdf [file pgen.1008607.s010.pdf]

Revision of PGENETICS-D-19-0131(RHS-elements function as Type II Toxin-Antitoxin modules that regulate intra-macrophage replication of *Salmonella Typhimurium*)

Dear Reviewers,

We enclose a substantially revised version of our manuscript entitled *"RHS-elements function as Type II Toxin-Antitoxin modules that regulate intra-macrophage replication of Salmonella Typhimurium"*.

We thank the four reviewers for their useful and constructive comments, and have incorporated their suggestions and modified the manuscript as outlined in detail below. This includes presentation of new data in main figures 3, 4, and 6 and supplemental figures S3 and S5. In addition, we have substantially rewritten the manuscript to make it easier to understand and added to the discussion of the results according to the excellent suggestions made by the reviewers. Please find point-by-point responses to the reviewer's comments attached.

We hope that these changes make the manuscript acceptable for publication. We feel that we have addressed all of the reviewer's concerns and hope that this new version will merit acceptance for publication in PLOS genetics.

Best regards,
/Sanna Koskiniemi

Point-by-point response to reviewer comments

Reviewer #1:
The manuscript from Hammarlöf et al. describes functional analysis of Rhs toxin systems from Salmonella Typhimurium. The Rhs locus contains two sets of toxin and inhibitor-coding ORFs. Internal promoters are shown to allow expression of these two pairs, suggesting their activity as type II toxin-antitoxin systems. Deletion analysis of Rhs locus demonstrated that these toxin pairs reduce growth in macrophages.

This is an interesting study which blends the fields of protein toxin secretion and internal toxin-antitoxin systems. On the whole, the data are well presented. There are, however, multiple unresolved questions and the current claims require further support before these two systems can truly be considered toxin-antitoxin systems.

Major points:
1) The assays of Figures 3 and S3 are poor and do not clearly demonstrate the extent of toxicity nor reproducibility. As the authors have the strains at hand, I would strongly recommend that the assays are repeated as full viable count assays using vector only controls where appropriate.

-Excellent suggestion. Fig 3 and S3 have been changed and now show toxicity of expressing the Rhs-main or Rhs-orphan toxin as cfu counts.

2) To be considered type II toxin-antitoxin systems, the authors should demonstrate (i) an interaction between the two components and (ii) whether or not the antitoxin and/or TA complex perform transcriptional autoregulation, as per other TA pairs. Perhaps the first point has been published in previous Rhs literature?

-We agree that to be considered type II toxin-antitoxins a direct interaction between the toxin and antitoxin should be demonstrated. We have attempted to do so by expressing HA-tagged Rhs-orphan immunity in the presence and absence of toxin and Formaldehyde cross-linking. Formaldehyde provides strong cross-linking that will not be denatured by SDS. Therefore, a clear shift in protein size of the tagged Rhs immunity can be observed using western blot with anti-HA antibodies (Fig. 4). In addition, we used magnetic beads with anti-HA antibodies to pull-down the orphan Rhs immunity in the presence or absence of Rhs orphan toxin. Using anti-Rhs-orphan antibodies we show that Rhs-orphan toxin co-purifies with Rhs orphan immunity in the presence or absence of cross-linker, suggesting a direct interaction between toxin and antitoxin (Fig. 4). This is discussed on page 8, line 184 and forward.

Although autoregulation has been demonstrated for a number of type-II TA systems, this is absolutely not the case for all systems and we do not see that as a prerequisite for a system to be considered a type-II TA system. Even though we find the regulation of Rhs expression very interesting and intend to follow up on it, we consider this to lay outside of the current work.

3) The toxicity assays demonstrate that rhs-orphan is not toxic. Yet, the Rhs-orphan mutant has the biggest impact in the macrophage assays, allowing the mutants to replicate. This implies Rhs-orphan is in fact toxic under certain conditions perhaps? Could this be addressed in the discussion?

-The Rhs-orphan is toxic, although to a lesser extent that the Rhs-main toxin, which should be clear from the new data in figure 3. We have included a section discussing the discrepancy between toxicity and effect on page 17, line 394 and forward.

4) Can the authors speculate further about toxin targets, from modelling or database searches?

- We have performed structure modelling and function predictions using (iTasser, Phyre, PFAM) and none of our searches gives any indication of the toxin function or target for the Rhs main toxin. The Rhs orphan toxin is a predicted to contain a Ntox47 domain by PFAM. This domain is found in RNase toxins from bacterial polymorphic toxin systems. This is now mentioned in the discussion on page 17, line 403 and forward.

5) Can the authors discuss in further detail regarding the lack of phenotype for rhs-complete in figure 5C?

We have corrected the order of the graphs in Figure 5 and the description in the text page 11, line 264 and page 12, lines 277-279 should now match the figure. The rhs-complete mutant has an additional phenotype to the mutant lacking the two toxins. Possible reasons for this are discussed on page 16 line 372 and forward.

Minor points:
1) Line 93, missing word

-This sentence (Page 4, line 88-90) has been rephrased and now reads: *"Effectors can be delivered through the needle-tube or by attachment to the tip of the puncturing device. The tip of the T6S devise is formed by e.g. the PAAR-domain containing Rhs toxins"*

4) Line 211 missing superscript.

-This has been changed.

5) Figures 5D and 5C have been swapped over in the text/figure, they do not match (lines 227-233.

-This has been amended.

6) Figure 5C (as on figure), the y axis is labelled oddly – what does it mean?

-Agreed, the Y axis has been changed to now show relative fold replication. The term is explained in detail on page 11, line 271.

7) Figs 5B-D, lighten shading of bars to make error bars more visible.

-Excellent suggestion, this has been amended.

Reviewer #2:

This work describes the expression and the role of Rhs toxins, namely Rhs-CTmain and Rhs-CTorphan, in Salmonella Typhimurium during macrophage infection. These Rhs toxins are in the type II toxin-antitoxin (TA) system and also exhibit contact-dependent growth inhibition (CDI). The authors first identify the internal promoters of the rhs genes and then demonstrate that the two toxins can be expressed from those regions, which is one of the characteristic traits of the type II TA modules.

Then, they confirm that the internal transcription yields the toxins by expressing them in Escherichia coli under arabinose-inducible promoter. The bacteria exhibit a growth defect on LB agar plates with L-arabinose only when the region that encodes the cognate immunity protein is not included. The authors also measure the levels of proteins produced from the internal transcripts by inserting YFP downstream of the start codon and using fluorescence microscopy to determine the YFP signal as a read-out. In addition, the authors demonstrate that the cognate immunity proteins are susceptible to degradation by a stress-induced protease, Lon and propose the mechanism how Rhs toxins become active inside macropahges. Finally, by using genetic knockouts and flow cytometry, their data indicate that the main role of Rhs toxins is to arrest growth of Salmonella upon entry into macrophages.

Overall, these findings explain the expression data from the Hinton Lab (Srikumar et al., PLoS Pathog., 2015) why rhs genes are up-regulated when bacteria are inside macrophages. The experiments are well-designed. Although the finding that Rhs toxins seem to be important in intracellular replication, many of the other findings are not all that new and surprising.

Major Comments:
1. In Figure 3 under the rhs-CTmain ORF1(CTG) column (left column), why does the E. coli strain without any plasmid not grow on the plate in the presence of L-arabinose?

-The no plasmid refers to no immunity plasmid. The strain expressed the toxin in the absence of immunity resulting in toxicity. As the empty pBR322 plasmid (the backbone used for cloning Rhs-main and Rhs-orphan immunites provides resistance to both ampicillin and tetracyclin (whereas the versions with immunity only provide resistance to ampicillin as the immunity gene is cloned in place of the tetracyclin gene) we cannot use an empty plasmid for this experiment. Fig 3 and S3 have been changed and now show toxicity of expressing the Rhs-main or Rhs-orphan toxin as cfu counts and the label has been changed to no immunity, which we hope is easier to understand.

2. The authors propose that the cognate immunity proteins of Rhs toxins are degraded by Lon, which is also up-regulated inside macrophages. If that is the case, how do the bacterial cells that express Rhs toxins remain viable once the cognate immunity proteins are degraded? What happens in individual Salmonella cells with regard to Lon expression and degradation of immunity proteins to Rhs? Doesn't Lon degrade many anti-toxins?

-This is an interesting thought and we have included a short section in the discussion about this, page 17, line 390 and forward.

3. Following up from question 2, why does Salmonella have both Rhs-CTmain and Rhs-CTorphan if they are mainly used to control the intracellular growth of the pathogen?

-Excellent point. We have included a discussion of this on page 11, lines 322 and forward.

4. Rhs toxins seem to have multiple roles. The previous work done by Koskiniemi (PLOS genet., 2014) suggest that Rhs toxins are used for a clonal selection. Although it is already stated in the discussion, it might be worth having a small paragraph to discuss the two studies more clearly somewhere.

-Good suggestion, we have expanded the section in the discussion regarding this, Page 11, line 322 and forward.

5. Could the authors speculate why these Rhs toxins are also expressed in some other conditions like in early stationary phase (ESP)?

-The toxins are probably expressed in all conditions to avoid being lost, which could explain why Rhs genes are under such high positive selection. This is discussed in detail in the discussion page 15, lines 341-344.

6. Are the Rhs toxins mediating contact-dependent killing of sister cells within the macrophage?

-It is possible that the differences observed between the strain lacking the two Rhs toxins or the entire *rhs* locus, arise from the ability of cells to deliver toxins in the macrophage. We have included a section where we discuss this on page 16, line 372 and forward.

Minor Comments:

1. From line 225 onwards, please check if the Figure 5C and 5D are swapped. The description in texts is not matched with the data present in each figure.

We have corrected the order of the graphs in Figure 5 and the description in the text page 11, line 264 and page 12, lines 277-279 should now match the figure.

2. Line 422, should the word "YFP" be replaced with GFP or FITC? Or did the authors actually use the setting for YFP to gate the GFP signal from the pDiGc plasmid?

-This has been corrected.

Reviewer #3:
In this manuscript he authors demonstrate that Rhs elements in S. Typhimurium function as *toxin-antitoxin systems and contribute to growth regulation in macrophages. They present a possible model for how the Rhs do this by demonstrating that their activity is regulated by a protease known to be expressed during intracellular growth. The experimental plan and conclusions are overall very solid. Below are some comments:*

For Figure 4, I'm not sure I understand what is being shown. For one, there is a lot more than 6-fold more expression with ATG instead of CTG. I'm not convinced that you're actually measuring the protein production level of the native gene using this construction. If you wanted to actually measure the protein production level, you should make an inactivating mutant in the toxin then use a quantitative western or something like that. You know that the promoter is active. Regardless of how much is produced, you know that enough toxin is made to restrict growth of E. coli. You aren't accurately quantifying protein production levels, so this figure doesn't add anything.

-Agreed. We have removed the data where the start codon has been replaced and shortened this section in the manuscript to simply say that there is translation occurring from the internal promoter. The figure has been moved to the supplement (Fig. S4).

For the text discussion of figure 5, I was really confused... I think the references to figure 5c and 5d are switched? Anyway, the biggest difference between mutants in 5b is between the 3rd and 4th columns. The authors seem to be suggesting that this difference is explainable by differences in different delays in replication starting, but the bar graphs don't really show that – the ones with more survival have a longer delay before starting replication. Maybe I just don't understand how the measurements are being made. Maybe the authors could reword this paragraph to make it more clear.

- Thank you for pointing this out. We have corrected the order of the graphs in Figure 5 and the description in the text page 11, line 264 and page 12, lines 277-279 should now match the figure. We have also rewritten the entire section regarding the infection experiments (pages 11-12) in order to make it easier to understand.

I couldn't help but notice that the authors avoided commenting on differences between Main and Orphan in their data. For example, in fig 6, it seems like lon has a much greater impact on Orphan than the main cluster. Similarly, in Figure 4, there are some differences between Main and and Orphan. Can anything be concluded about the roles of the two CTs?

-Good point. We have included a section in the discussion about the difference between the two toxins on page 14, line 322 and forward, page 17 line 394 and forward.

Reviewer #4:
In this manuscript, Hammarlöf et al. describe RHS toxins of Salmonella that instead of being delivered could serve as intrabacterial toxin, thereby regulating proliferation during macrophage infection. They show that rhs ORFs contain internal promoters that could result in the production of toxic proteins, whereas the delivery part of the system is not expressed, suggesting internal intoxication is possible. Then, they show that deletion of rhs toxin systems affects growth rate or proportion of growing bacteria during the course of macrophage infection. Finally, they provide supporting evidence that Lon is the protease that might participate in the activation of the intoxication upon degradation of the immunity protein.
Most of experiments are carefully carried out and results are interpreted cautiously. The results are very interesting and open the way for consideration of the role of many other toxin systems in self-controlling bacterial physiology rather than compete with neighbours.

Main comments:
Figure 1: What is the timepoint of the Raw infection? A time course would be valuable in the sense that it would strengthen the observation of expression during infection and inform on when the systems are activated.

-Bacteria were harvested from RAW cells after 8h of infection. This is stated in the results and methods section pages 6, lines 138-139 and pages 24, line 555 as well as in the figure legend. Considering the very small effect, one addition doubling, that we see in our infection experiment, it is highly unlikely that we would see any effect of the Rhs toxins earlier in the experiment as the main effect is on the growth rate of the bacteria.

Figure 2: Overnight should be defined to allow reproducibility of the data. The activity of P1 and P3 is hard to see whereas the statistical tests suggest it is present. The authors must revisit the stats or provide evidence for activity. There seems to be heterogeneity of the promoter activity (P2), the authors could elaborate on that.

-Thank you for pointing this out. Overnights are now defined as (16-18h) on page 7, line 153. The activity of P1 and P3 are not over baseline (hatched line) and cannot be considered biologically relevant, although very little variation between biological replicates results in statistical significance. The population with no expression seen in the old experiment represents dead bacteria (which have lost the pEH167 plasmid and died do to the antibiotic in the media). When we repeated the experiment in a strain expressing dTomato from the chromosome, this population is no longer present.

Figure 3: The authors prove that the genes encode toxic proteins that could be expressed of P2 and neutralised by their cognate immunity proteins. Could they provide toxicity/immunity quantification during liquid culture? As a minor comment, the figure should be labelled for "toxins" in X axis and "immunity" in Y. The nomenclature is not very intuitive and if the authors cannot rename the different genes, they should help readers identify easily what is what.

-Excellent suggestion, we have repeated the experiment and now present toxicity of the two toxins in terms of cfu values. The entire figure has been regraphed.

Figure 4: The title of the figure should be rephrased. This figure should be revisited, and the authors should use Flow Cytometry as opposed to microscopy to quantify fluorescence. Fluorescence of Rhc-CTorphan CTG-YFP is too weak to be convincing, therefore the microscopy images have limited interest. The authors should discuss the implications that they have to switch the start codon to detect protein production. What does that mean for the endogenous levels of toxins being produced?

-The signal is too low to be detected by flow cytometry (which requires ~500 YFP molecules /cell for signal). We agree that the data is not convincing and does not add much to the paper. We have removed the data where the start codon has been replaced and shortened this section in the manuscript to simply say that there is translation occurring from the internal promoter. The figure has been moved to the supplement (Fig. S4).

Figure 5: 208-209: what do you mean by "growth inhibition"? 214 "dormant" should be changed for "non-growers". Why have the strains been mixed for infection? Or it this a mistake in the text? How do you differentiate them? Can you run similar experiments with strains compared in parallel? It seems particularly important with the differential effect observed for the different mutants, for example the rhs complete deletion might not yield any phenotype. Authors should complement their mutants that have a phenotype. Panel C and D are switched. Authors should state in the text that although not significant there is a trend in increased intra-macrophage survival for each mutant, otherwise it is very hard to relate survival, start of replication and growth rate. I would accept that the overall survival is a much cruder measure so it is much harder to get significant results, but this needs to be acknowledged for the results to make sense.
"starts replication" is confusing. Do the authors mean "proportion of growers"?

-Excellent suggestions. We have corrected the order of the graphs in Figure 5 and the description in the text page 11, line 264 and page 12, lines 277-279 should now match the figure. We also discuss the effect of each mutation on proliferation within macrophages more in detail on page 14. The effect of lacking both Rhs toxins is very small, resulting in one addition doubling, during the 15h time-frame of the infection experiment. Even though seeding each well with the same amount of RAW 264.7 cells, the actual number of cells at the beginning of the experiment (24h later) easily varies with a factor of two. To be able to get statistically significant data, we had to remove this variable from our experimental set up. To this end, we chose to infect RAW cells with a mixture of wild type and mutant bacteria. Using a low MOI, we ensure that only one bacteria will be taken up in each macrophage. By having different antibiotic markers in the wild type and mutant bacteria allow us to separate the two bacteria and calculate growth/survival of the wild type and mutant separately. We have tried to clarify this in the text on page 9, line 222 and forward.

"dormant" has been changed to "non-growers"
"start replicating" has been changed to "proportion of growers".

Figure 6: Panel A – I do not agree with the conclusion that rhsImain is more stable in a lon mutant seeing the variation of the loading control in the wt background. Other blot with a stable loading control must be provided or quantification over several repeats.

We have repeated the western blot in Figure 6 with adjusted levels of sample to account for the general degradation effect of the active Lon protease. We agree that Lon does not seem to degrade *rhsI* and have rephrased the conclusions in the text accordingly: "*The RhsI^{orphan} protein was stable over the course of 1h in Δlon bacteria compared to wild type where degradation occurred already after 15 min after SHX addition (lower panel). In contrast, the RhsI^{main} protein was degraded 30 min after SHX addition in both wild type and Δlon bacteria, suggesting that another protease is responsible for the degradation of this protein (upper panel) (Fig. 6A).* ", page 13, line 298 and forward.

Minor comments:

L177 – L-Arabinose plates

-This has been changed.

Figure 5 C and D must be bold

-This has been changed.

L263: "Rhs immunity proteins do"

-This has been rephrased.

L268 – please tone down

- Agreed. This sentence has been rephrased and now reads: *"Here we show that Rhs toxins are not only being delivered between cells but are also expressed and functional within a bacterial cell."*

Two papers cited in the references have been retracted and should be replaced by others.

-These references have been removed.
