# Supplementary material for: RHS-elements function as type II toxin-antitoxin modules that regulate intra-macrophage replication of Salmonella Typhimurium
Source: PLoS Genet. 2020 Feb 13;16(2):e1008607. doi: 10.1371/journal.pgen.1008607 (PMC7043789; doi:10.1371/journal.pgen.1008607)
Supplement: Attachment — Submitted filename: Hammarlof, Starsta et al., 2019 reviewing SH.docx [file pgen.1008607.s009.docx]

***RHS-elements function as Type II Toxin-Antitoxin modules that regulate intramacrophage replication of Salmonella Typhimurium***

***Hammarlöf D.L†, Stårsta M†, Wäneskog M, Xu F, Heden Gynnå A, Borg M, Herschend S and Koskiniemi S*.***

In this manuscript, Hammarlöf *et al*. describe RHS toxins of Salmonella that instead of being delivered could serve as intrabacterial toxin, thereby regulating proliferation during macrophage infection. They show that rhs ORFs contain internal promoters that could result in the production of toxic proteins, whereas the delivery part of the system is not expressed, suggesting internal intoxication is possible. Then, they show that deletion of rhs toxin systems affects growth rate or proportion of growing bacteria during the course of macrophage infection. Finally, they provide supporting evidence that Lon is the protease that might participate in the activation of the intoxication upon degradation of the immunity protein.

Most of experiments are carefully carried out and results are interpreted cautiously. The results are very interesting and open the way for consideration of the role of many other toxin systems in self-controlling bacterial physiology rather than compete with neighbours.

Main comments:

Figure 1: What is the timepoint of the Raw infection? A time course would be valuable in the sense that it would strengthen the observation of expression during infection and inform on when the systems are activated.

Figure 2: Overnight should be defined to allow reproducibility of the data. The activity of P1 and P3 is hard to see whereas the statistical tests suggest it is present. The authors must revisit the stats or provide evidence for activity. There seems to be heterogeneity of the promoter activity (P2), the authors could elaborate on that.

Figure 3: The authors prove that the genes encode toxic proteins that could be expressed of P2 and neutralised by their cognate immunity proteins. Could they provide toxicity/immunity quantification during liquid culture? As a minor comment, the figure should be labelled for “toxins” in X axis and “immunity” in Y. The nomenclature is not very intuitive and if the authors cannot rename the different genes, they should help readers identify easily what is what.

Figure 4: The title of the figure should be rephrased. This figure should be revisited, and the authors should use Flow Cytometry as opposed to microscopy to quantify fluorescence. Fluorescence of Rhc-CTorphan CTG-YFP is too weak to be convincing, therefore the microscopy images have limited interest. The authors should discuss the implications that they have to switch the start codon to detect protein production. What does that mean for the endogenous levels of toxins being produced?

Figure 5: 208-209: what do you mean by “growth inhibition”? 214 “dormant” should be changed for “non-growers”. Why have the strains been mixed for infection? Or it this a mistake in the text? How do you differentiate them? Can you run similar experiments with strains compared in parallel? It seems particularly important wrt the differential effect observed for the different mutants, for example the rhs complete deletion might not yield any phenotype. Authors should complement their mutants that have a phenotype. Panel C and D are switched. Authors should state in the text that although not significant there is a trend in increased intra-macrophage survival for each mutant, otherwise it is very hard to relate survival, start of replication and growth rate. I would accept that the overall survival is a much cruder measure so it is much harder to get significant results, but this needs to be acknowledged for the results to make sense.

“starts replication” is confusing. Do the authors mean “proportion of growers”?

Figure 6: Panel A – I do not agree with the conclusion that rhsImain is more stable in a lon mutant seeing the variation of the loading control in the wt background. Other blot with a stable loading control must be provided or quantification over several repeats.

L263: “Rhs immunity proteins do”

Minor comments:

L177 – L-Arabinose plates

Figure 5 C and D must be bold

L268 – please tone down

Two papers cited in the references have been retracted and should be replaced by others.
